# Resonance states and beating pattern induced by quantum impurity scattering in Weyl/Dirac semimetals

**DOI:** 10.1038/srep36106

**Published:** 2016-11-03

**Authors:** Shi-Han Zheng, Rui-Qiang Wang, Min Zhong, Hou-Jian Duan

**Affiliations:** 1 Guangdong Provincial Key Laboratory of Quantum Engineering and Quantum Materials, School of Physics and Telecommunication Engineering, South China Normal University, Guangzhou 510006, China

## Abstract

Currently, Weyl semimetals (WSMs) are drawing great interest as a new topological nontrivial phase. When most of the studies concentrated on the clean host WSMs, it is expected that the dirty WSM system would present rich physics due to the interplay between the WSM states and the impurities embedded inside these materials. We investigate theoretically the change of local density of states in three-dimensional Dirac and Weyl bulk states scattered off a quantum impurity. It is found that the quantum impurity scattering can create nodal resonance and Kondo peak/dip in the host bulk states, remarkably modifying the pristine spectrum structure. Moreover, the joint effect of the separation of Weyl nodes and the Friedel interference oscillation causes the unique battering feature. We in detail an- alyze the different contribution from the intra- and inter-node scattering processes and present various scenarios as a consequence of competition between them. Importantly, these behaviors are sensitive significantly to the displacement of Weyl nodes in energy or momentum, from which the distinctive fingerprints can be extracted to identify various semimetal materials experimentally by employing the scanning tunneling microscope.

Progress in material preparation and experimental techniques has led to a surge of interest in two-dimensional (2D) Dirac materials such as graphene and surface states of topological insulators. Very recently, this concept is extended to 3D systems, known as topological Dirac semimetals (DSMs), which are newly-discovered bulk analog of graphene as a new topological states of matter. Recent experiments have identified a class of materials^1^,^2^,^3^ (Bi_1−*x*_In_*x*_)_2_Se_3_, Na_3_Bi, and Cd_3_As_2_ to be the DSMs. In these new Dirac materials, 3D massless Dirac fermions are excited around the doubly degenerate Dirac cones, which are protected by time-reversal symmetry (TRS) or inversion symmetry (IS).

Breaking either the TRS or the IS will drives the DSMs into a Weyl semimetal (WSM) phase, which is manifested as the splitting of a pair of degenerate Weyl nodes with opposite chirality in momentum or energy space. As a new topological nontrivial phase, these massless WSM fermions are drawing great interest for their scientific and technological importance. The WSM Fermion states have been predicted theoretically and observed experimentally in a family of the noncentrosymmetric transition-metal monosphides^4^,^5^,^6^,^7^,^8^,^9^,^10^ with preserving the TRS, e.g., TaAs, NbAs, NbP, and TaP. The nontrivial topology along with the node separation leads to many exotic phenomena and unique physical properties, such as the chiral anomaly^11^,^12^,^13^, the unique Fermi arc surface states^6^,^7^,^8^,^9^,^10^,^14^, the chiral Hall effect^13^,^15^, the chiral magnetic effects^11^,^16^, and the negative^17^,^18^,^19^,^20^ and extremely large magnetoresistance^21^.

When most of the previous studies concentrated on the clean host WSM bulk states, it is expected that the dirty WSM system would present rich physics due to the interplay between the WSM Fermion states and the impurities embedded inside these materials. On one hand, the unique 3D spin-momentum locking can mediate the interaction between magnetic impurities in both Dirac and Weyl semimetals, leading to anisotropic Ruderman-Kittel-Kasuya-Yosida (RKKY) coupling and rich spin textures^22^,^23^,^24^. On the other hand, the feedback effect of impurities on host bulk states can change the property of Weyl nodes, at which the differences between a Weyl phase and a normal metal are most pronounced. The stability of the nodal density of states (DOS) was investigated in the presence of various types of local impurities^25^,^26^, and nonzero DOS at the degeneracy point was predicted for the disorder strength beyond a certain critical value^27^,^28^. We would like to mention that these discussions, however, are limited to the classic impurity model and only the single node scattering is taken into account. The nodal resonance induced by impurities have also been extensively studied in graphene and topological insulators^29^,^30^,^31^,^32^,^33^. As a representational feature of quantum impurities, the Kondo effect has been intensely discussed in three-dimensional Dirac and Weyl systems^34^,^35^,^36^,^37^ of dilute magnetic impurities. The results showed that the nature of the Kondo effect of impurity is only affected strongly by the linear dispersion of Dirac/Weyl host bulk states but it is in general blind to the momentum splitting of TRS-broken Weyl nodes. In ref. 35, they found that the spatial spin-spin correlation between the magnetic impurity and the conduction electron is sensitive to the displacement in the momentum splitting of Weyl nodes, where rich features are shown due to an extra phase factor. Even so, it is still challenging how to identify the TRS-broken WSM materials from the transport fingerprints.

In this paper, we study how the local density of states (LDOSs) in host WSM/DSM bulk states are modulated by the embedded quantum impurity in the resonance regime and in the Kondo regime. We specially pay attention to the response of nodal behavior to impurity scattering processes. It is found that the quantum impurity scattering can create a LDOS resonance or Kondo peak/dip in the host bulk states exactly at the Dirac point and thus remarkably destroy the pristine spectrum structure, which are sensitive to the degree of the splitting of two WSMs nodes. Compared with the single node scattering, the internode scattering possesses more information about the unique properties. Interestingly, by taking the intranode scattering into account, we find the unique battering feature for the TRS-broken WSMs, which is long-range measurable in real space with current scanning tunneling microscope technologies.

The rest of the paper is organized as follows. In Sec. II we present a general interaction model of Weyl fermions with Anderson quantum impurity and treat it by employing the standard equations of motion for Green’s functions. The low-energy resonance, Kondo signature, and Friedel oscillation in host materials are discussed in Sec. III, and a short summary is given in the last section.

## Model and Theory

Consider a 3D WSM with a pair of chirality-opposite Weyl nodes, whose low-energy Hamiltonian can be described as^22^,^34^ 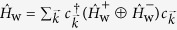, with


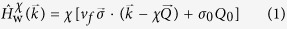


and the annihilation operator of electrons 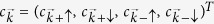 acting on the spin and chirality spaces. Here, *χ* = ±1 represents the pair of weyl nodes with the opposite chirality, *v*_*f*_ is the Fermi velocity, 
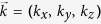
 is the effective wave vector measured from the Weyl nodes, and 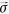 denotes the vector of Pauli matrices, For 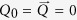, 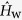 reduces to the Hamiltonian of degenerate DSM, possessing both the TRS 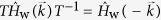 and the IS 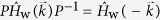, where 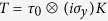 with complex conjugation operation *K* is the time-reversal operator, *P* = *τ*_*x*_ ⊗ *σ*_0_ is the inversion operator, and 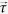 is the Pauli matrix on the chirality space. Breaking either TRS 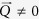 or IS *Q*_0_ ≠ 0 transforms a DSM into a Weyl system, the former splitting the two degenerate weyl points separately at different momentum 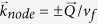 but with the same energy while the latter shifting two Weyl nodes at different energy *ω*_*node*_ = ±*Q*_0_ but with the same momentum. This can be seen from the dispersion spectrum of 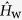,


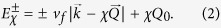


We utilize the typical Anderson impurity model to study the quantum impurity effect and spin-1/2 Kondo screening in 3D Dirac and Weyl semimetals. The full Hamiltonian can be written as 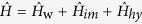. The impurity Hamiltonian


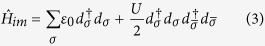


is characterized by a single-orbital energy *ε*_0_ and the on-site Coulomb repulsion *U*. 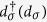 is the creation (annihilation) operator for impurity electrons. 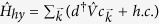 represents the hybridization between the impurity and the host material with the hybridization matrix


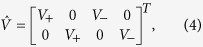


where the spinor 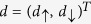 and the coupling strength *V_x_* is assumed to be dependent on the Weyl node *χ* but regardless of 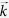 and 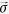 under the assumed wide-band approximation and spin-conserved hoping. Here, we also assume that the magnetic impurities are embedded inside the WSM such that the effect of Fermi arc surface states can be neglected safely.

Using the method of standard equations of motion, the retarded Green’s function of Weyl electrons with respect to the full Hamiltonian 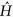 can be derived as 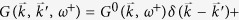 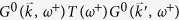, where *ω*^+^ = *ω* + *i*0^+^ and all qualities are 4 × 4 matrix in the spin ⊗ chirality space. When performing the Fourier transform to the real space, its block matrix in chirality space is


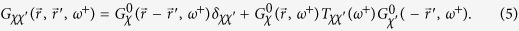


The Green’s function 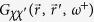 is still 2 × 2 matrix in subspace of the electron spin and 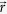 is measured from the impurity as a scattering center, whose position is chosen to be the origin of coordinates. The expression in Eq. (5) recalls the extensively applied T-matrix approach^30^,^31^, but here 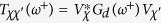 is expressed in terms of the Green’s function of magnetic impurity, defined as 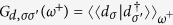, which is the Fourier transform of 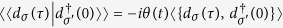. Similar relation can be found in Anderson impurities interacting with topological insulator^29^,^33^,^38^,^39^ or graphene^40^. In Eq. (5), 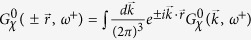 is the bare Green’s function of Weyl fermions with 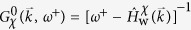. At the impurity position 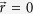, the bare Green’s function is given by


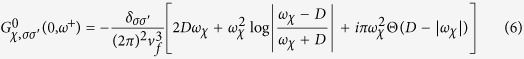


with the cutoff energy *D*, unitstep function Θ(*x*), and *ω*_*χ*_ = *ω*^+^ − *χQ*_0_. Note that even for STR-broken case due to finite 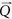, 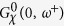 is diagonal in spin space and independent of 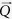, remarkably different from the case of 2D topological insulator or graphene^33^,^41^. For 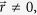 we can calculate 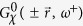 by expanding the 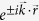 in terms of spherical harmonics according to the Rayleigh equation^24^,^42^, and finally arrive at a simple analytical expression for *D* ≫ *ω* as


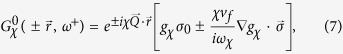


where we define 
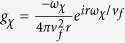
 and 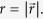


The next task is to calculate the impurity Green’s function 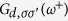. Carrying out the equations of motion, we find


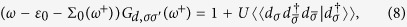


with the retarded self-energies 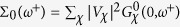. Further calculation gives


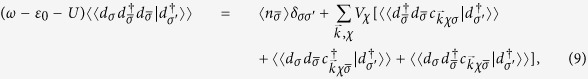


where three high-order Green’s functions emerge. Performing the same procedure with the equation of motion, we obtain all high-order Green’s functions and in the following take 
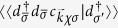
 as an example,


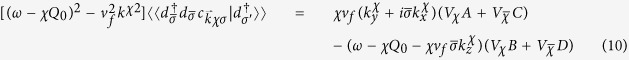


with


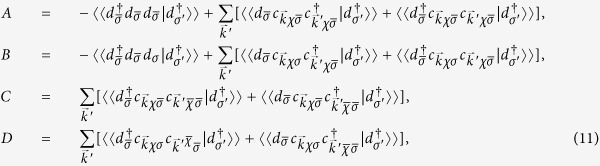


and 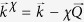, where we denote 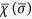 opposite to *χ*(*σ*). To form a set of close iterative equations, we truncate them following the standard method^43^, for example, 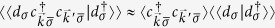, where the operator pair with the same spin indices can be pulled out of the Green’s function as an average and is calculated with the Fluctuation dissipation theory


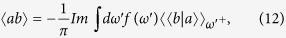


where *f*(*ω*) is the fermi distribution function. After carrying out lengthy but straightforward calculations, we finally derive the expression for the impurity Green’s function in the deep Coulomb blockade regime, i.e., *U* → ∞, as


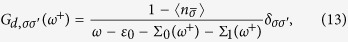


with


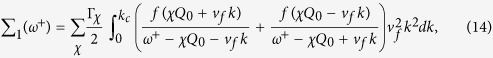


where the coefficient 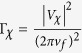. By comparison with the normal metals^44^ or 2D Dirac materials^33^, the most distinction is the specific expressions of self-energies ∑_0_(*ω*^+^) and ∑_1_(*ω*^+^).

## Results and Discussion

### Resonance states in LDOS

Our purpose is to explore the unique local properties of the WSM when the conducting elections are scattered off a quantum impurity. As the Weyl nodes are separated in energy or momentum, a very interesting question is whether 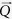 or *Q*_0_ leads to some especial spectrum structures locally around the quantum impurity. Next, we focus on the LDOS in WSMs, which is defined as


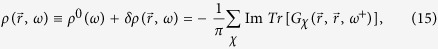


where 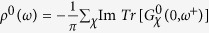 is the unperturbed LDOS, and 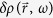 contributed by the second term in Eq. (5), reflects the substantial modification of the LDOS by the doping impurity. Beyond the usual single-node treatments, we here emphasize the impurity scatter processes between two Weyl nodes. We find that the introduce of quantum impurity not only scatters the electrons within the same Weyl node but also between two nodes. Specifically, we can split LDOS as 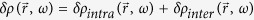, where 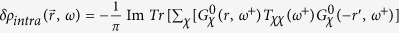 collects the contribution from the intranode scattering process, equivalent to single-node situation, while 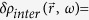 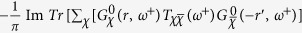 collects the contribution from scattering process between two nodes. After proceeding the calculations, we obtain readily the following analytical expressions


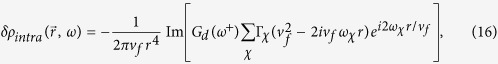


and


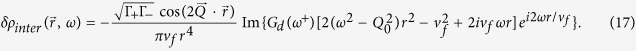


**Figure 1:**
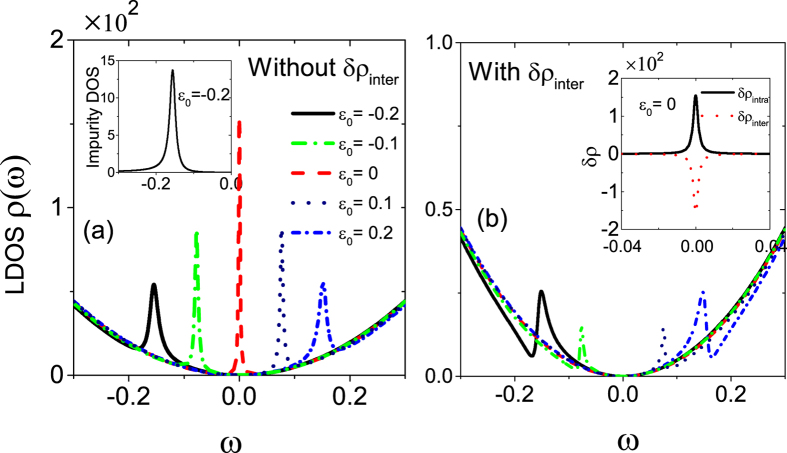
The low-energy resonance near the quantum impurity in LDOS for DSM materials (*Q*_0_ = *Q*_*z*_ = 0). The total LDOS 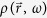 (**a**) without and (**b**) with the internode scattering for different impurity levels *ε*_0_ as indicated. Inset in (**a**) is the impurity DOS and inset in (**b**) is the correction of LDOS 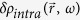 and 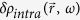. The chosen other parameters are Γ_+_ = Γ_−_ = 0.05, *r* = 4, *T* = 10^−5^, *v*_*f*_ = 1. All energies are in unit of the cutoff energy *D*.

Equations (16) and (17) are our central results. In order to understand them deep, we in the following limit our discussions to the symmetrical coupling Γ_+_ = Γ_−_, and first discuss the impurity effect in the DSMs, i.e., setting 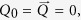 whose LDOS *ρ*(*ω*) for a fixed 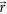 is illustrated in Fig. 1. Without the internode scattering (i.e., single-node case), seeing Fig. 1(a), there a pronounced resonance structure, whose position depends on the impurity level *ε*_0_. This resonance is a consequence of the backaction of the resonance in the impurity DOS, which is defined as 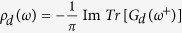 and depicted in the corresponding inset, indicating the single-level resonance tunneling between the impurity and the reservoirs. In Fig. 1(a), with the increase of *ε*_0_ from −0.2 to 0.2 in step of 0.1 the low-energy resonance is first shifted close to the Dirac point, accompanied with increasing magnitude, and then passes over the Dirac point into its other side, on whole exhibiting a symmetry with respect to the Dirac point. Intriguingly, a sharp pronounced resonance for *ε*_0_ = 0 can be located exactly at the Dirac point, completely destroying the 3D typical *ω*^2^ Dirac spectrum. Similar Dirac-point resonance appears in doping surface of topological insulators with quantum impurities^33^ or quantum magnets^44^,^45^. Our further calculations confirm that the scenario of Dirac-point resonance cannot emerge for classic impurity model, i.e., replacing 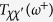 in Eq. (5) with 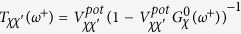, where 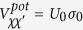 stands for a classic impurity potential. If the internode scattering is taken into account, the scenario is very different from the single-node case. We plot the LDOS *ρ*(*ω*) including both intra- and inter-node scattering in Fig. 1(b). By comparison with single-node case, most interesting in double-node case is that the resonance peak becomes weaker and weaker when close to the Dirac point and is completely smoothed away at the Dirac point, in which *ρ*(*ω*) ∝ *ω*^2^ recovers the typical square dependence on energy. To understand it, we plot the change of DOS *δρ*_*inter*_(*ω*) and *δρ*_*intra*_(*ω*) for *ε*_0_ = 0 in the inset of Fig. 1(b), from which we know that the negative *δρ*_*inter*_(*ω*) tends to suppress the resonance in *δρ*_*intra*_(*ω*) and, at Dirac point *ω* = 0 they have the same amplitude but opposite sign and thus cancel each other exactly. This point also can be seen from Eqs (16) and (17).

**Figure 2:**
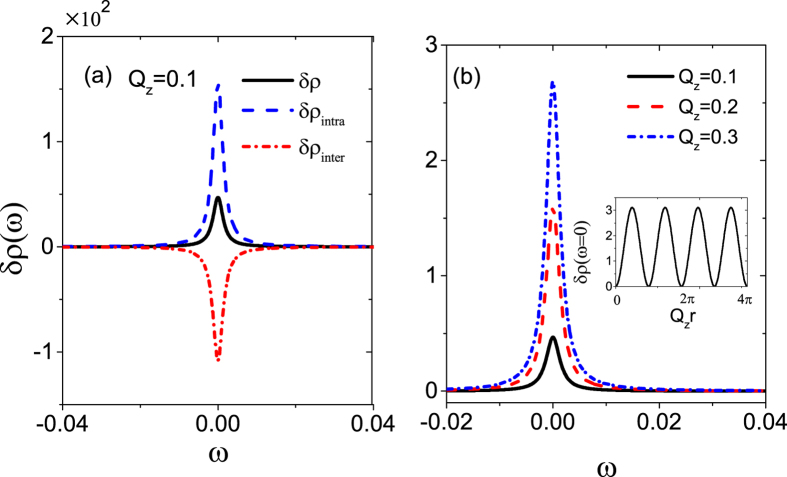
The low-energy resonance in LDOS for TRS-broken WSMs (*Q*_*z*_ ≠ 0, *Q*_0_ = 0). (**a**) The correction of LDOS 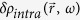 and 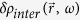 as a function of energy *ω*, and (**b**) the evolution of 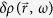 for different *Q*_*z*_ values. Inset: the periodic oscillation of 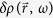 with *Q*_*z*_. The other parameters are the same as in Fig. 1.

From the above discussions for DSMs, we are known that the competition between intra- and inter-node scatterings is crucial for the development of the Dirac-point resonance. In Fig. 2(a) we depict the change of LDOS *δρ*(*ω*) for the TRS-broken WSMs, i.e., *Q*_0_ = 0 but 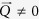. Here, we just choose 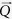 along *z*-axis and so the degenerate Weyl nodes are shifted by ±*Q*_*z*_ in the direction of 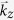 but *Q*_*x*/*y*_ = 0. Obviously, the Dirac-point resonance for finite *Q*_*z*_ recovers since *δρ*_*inter*_ only partly offsets *δρ*_*intra*_, as shown in Fig. 2(a). From Eqs (16) and (17), one can notice that 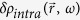 is independent of 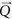 but 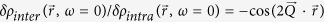 is less than −1 for the chosen parameter. The variation of LDOS 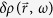 for different *Q*_*z*_ is plotted in Fig. 2(b), in which the Dirac-point resonance peak increases first for small *Q*_*z*_ ∈ (0, *π*/2*r*) and then exhibits a periodic function of *Q*_*z*_, seeing the inset. For *Q*_*z*_ = (2*n* + 1)*π*/4*r (n* = 0, 1 …) or 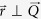, the internode scattering is prohibited due to destructive interference and thus 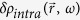 dominates. Therefore, to probe the feature of TRS-broken WSMs, it is necessary to consider the impurity-induced scattering between Weyl nodes since 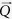 only enters *δρ*_*inter*_ but not *δρ*_*intra*_.

**Figure 3:**
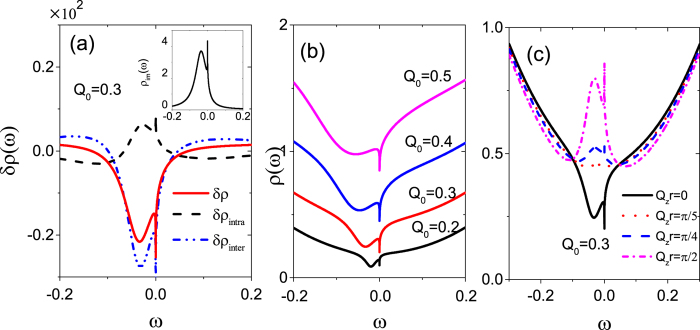
The Kondo resonance in LDOS for noncentrosymmetric WSMs (*Q*_0_ ≠ 0, *Q*_*z*_ = 0). (**a**) 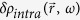, 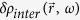, and 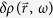 versus *ω* for *Q*_0_ = 0.3 and *Q*_*z*_ = 0, and the inset is the impurity DOS. The variation of the total 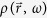 (**b**) for different *Q*_0_ = 0.2–0.5 in step 0.1 with *Q*_*z*_ = 0, and (**c**) for *Q*_*z*_ = 0, *π*/5, *π*/4, *π*/2 with *Q*_0_ = 0.3. Here *ε*_0_ = −0.01 and the others parameters are the same as in Fig. 1.

For noncentrosymmetric WSMs, i.e., *Q*_0_ ≠ 0 and 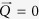, we from Eqs (16) and (17) see that *Q*_0_ contributes to both 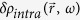 and 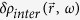 but with different ways, thus their zero-energy resonances cannot be completely compensated. Another most interesting effect for noncentrosymmetric WSMs is the emergence of Kondo resonance, which is expected to occur because of the nonzero LDOS at *ω* = 0 when two Weyl nodes are split to *ω*_*node*_ = ±*Q*_0_. If we choose the proper parameters in Kondo regime, the impurity DOS presents a remarkable sharp Kondo resonance at *ω* = 0 as shown in the inset. The Kondo resonance is mainly attributed to the self-energy 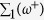 in Eq. (14), which depends on 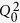 rather than linear *Q*_0_, distinct from graphene^40^ and topological insulator^41^. The results are in agreement with those obtained by number renormalization group^34^. Suffering from the scattering off the impurity potential, the electronic LDOS in the host semimetal material also exhibits the feedback of Kondo resonance in both 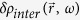 and 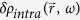. They have opposite sign but cannot compensate each other and so the total 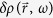 exhibits a dip structure as depicted in Fig. 3(a). We plot the evolution of the total LDOS 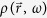 with *Q*_0_ in Fig. 3(b), where the Kondo dip becomes more and more prominent with the increase of *Q*_0_, companied by overall lift upwards due to the Weyl node pair shifting away from the zero energy. Interestingly, if we further consider a finite *Q*_*z*_, it will significantly reverse the Kondo structure from a dip to a peak, as illustrated in Fig. 3(c), as a consequence of the competition between two types of scattering processes. Similar to the Dirac-point resonance in Fig. 2(b), the evolution of Kondo peak from a dip to a peak is a periodic function of *Q*_*z*_, greatly different from the monotonously-increasing dependence on *Q*_0_. Note that the Kondo resonance develops only in the inverse-broken case with *Q*_0_ ≠ 0, which is a feature of the linear dispersion, similar scenarios appearing in TI or graphene^40^,^41^.

### Spatial Friedel oscillation of LDOS

In this section, we discuss the characteristics of Friedel oscillation, namely, the oscillation behavior of LDOS with the spatial distance 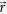 measured from the impurity position. This is caused by the interference of incoming and outgoing waves when conducting electrons are scattered off a local impurity potention. Since the dependence of LDOS 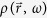 on 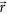 stems completely from the impurity scattering correction 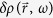, in following analysis we only focus on 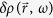.

**Figure 4:**
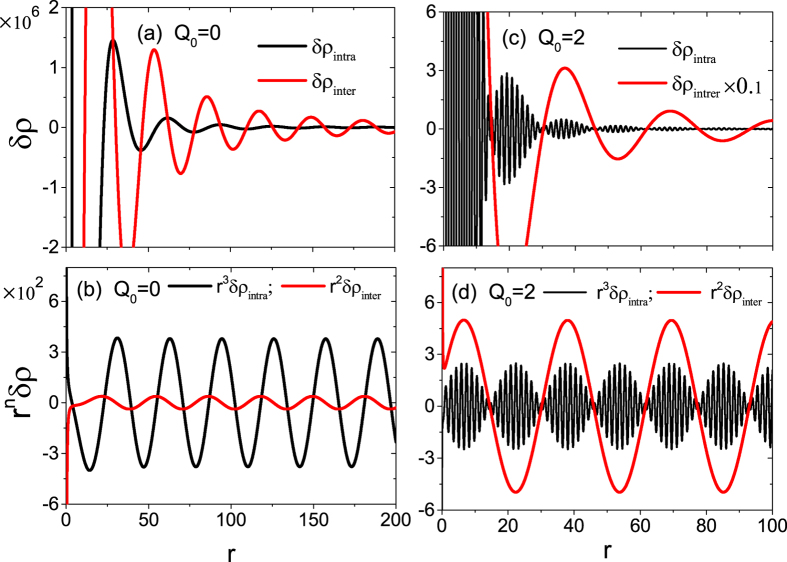
(**a**,**b**) The Friedel oscillations of 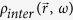 and 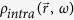 with distance *r* for the DSMs with *Q*_0_ = 0, and (**c**,**d**) the beating pattern for the noncentrosymmetric WSMs with *Q*_0_ = 2. In panels (**b**,**d**), 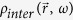 and 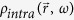 are scaled by *r*^2^ and *r*^3^, respectively. Here, *ε*_0_ = −0.01, *Q*_*z*_ = 0, *ω* = 0.1, and the others parameters are the same as in Fig. 1.

Figure 4(a) shows the variation of 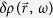 with 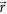 for the DSM materials (*Q*_0_ = *Q*_*z*_ = 0). Obviously, a typical pattern of Friedel oscillations is presented for both 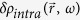 and 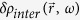. By comparison, the oscillation of 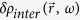 dominates in long distance while the oscillation of 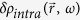 is in short distance. The reason is that the former decays as an inverse-square *r*^−2^ law and the latter as *r*^−3^ law, which can be seen from Fig. 4(b) where 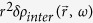 and 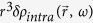 exhibit the equal amplitude oscillation. Figure 4(c,d) correspond to the case of noncentrosymmetric WSMs (*Q*_0_ = 2 and *Q*_*z*_ = 0). When 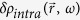 and 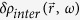 display a damped oscillatory behavior similar to *Q*_0_ = 0, there appears an interesting beating pattern in 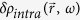. This beating feature is originated from the combination effect of the energy separation of Weyl nodes by ±*Q*_0_ and the Friedel oscillation, manifesting itself by the factors cos (2*Q*_0_*r*/*v*_*f*_)exp (2*iωr*/*v*_*f*_) and sin (2*Q*_0_*r*/*v*_*f*_)Exp (2*iωr*/*v*_*f*_), derived from Eq. (16). For 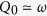, the beating feature vanishes and both 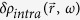 and 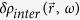 show 1/*r*^3^-law decaying oscillation. When two oscillating frequencies have distinct difference, the beating effect emerges, as illustrated in Fig. 4(c,d) where we choose *Q*_0_ ≫ *ω* and the length of beating is determined by 2*ω*. Inversely, the beating length is determined by 2*Q*_0_ for *ω* ≫ *Q*_0_. In real materials, it is reported *Q*_0_ = 23 meV for TaAs in ref. 6 and 36 meV for NbAs in ref. 7, which is within the range of low-energy spectrum due to usually *ħv*_*F*_ ≈ 0.37 eV and *D* ≈ 300 meV. Experimentally, the electron energy can be set to be larger or smaller than *Q*_0_ to observation both beating scenarios as discussed above. One, however, can notice that for *r* ≫ *v*_*f*_/*ω*, the long-range 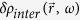 quickly dominates and is larger than the short-range 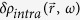 by at least one order in magnitude, which easily overwhelms this beat frequency in measurement of total LDOS. Therefore, to measure the *Q*_0_-induced beating structure, the electron scattering off the impurities must be limited to the same Weyl node.

**Figure 5:**
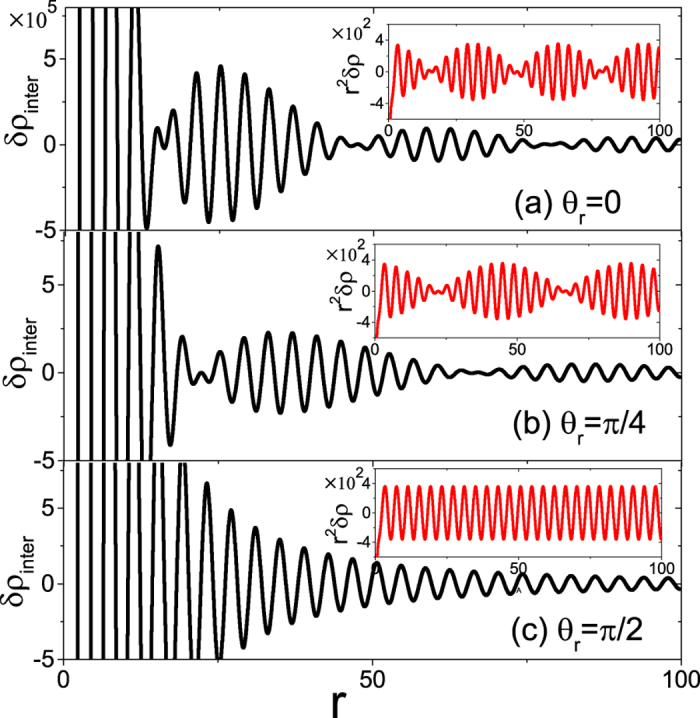
The dependence of beating pattern of 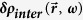 on the direction of 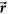 for TRS-broken WSMs. (**a**–**c**) panels are plotted for *θ*_*r*_ = 0, *π*/4, and *π*/2, respectively. Insets are 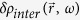 scaled by *r*^2^. All structures are independent of the azimuthal angle *φ*_*r*_. Here, *Q*_*z*_ = 0.05, *Q*_0_ = 0, *ω* = 0.8, *ε*_0_ = −0.01, and the others parameters are the same as in Fig. 1.

In contrast to the noncentrosymmetric WSMs, the nonzero 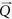 in TRS-broking WSMs adds an extra phase factor 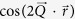 in 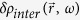 but has nothing to do with 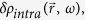 seeing Eqs (16) and (17). The displacement of the Weyl nodes in the momentum will further induce complexity to the Friedel oscillation behavior of 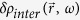. Similarly, there are two periods associated with 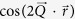 and 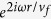, exhibiting a batter pattern for large difference 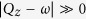, where we choose 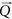 along *z*-axis and denote *r*_*z*_ = *r*sin *θ*_*r*_ with respect to the *z*-axis. Obviously, the beating characteristics is dependent on the spatial direction *θ*_*r*_ but independent of the azimuthal angle *φ*_*r*_, which is a consequence of the azimuthal symmetry around the correcting line of a pair of Weyl nodes (i.e., chosen *z*-axis). The spatial direction dependence of 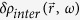 is plotted in Fig. 5(a–c) for *θ*_*r*_ = 0, *π*/4, and *π*/2, respectively. Figures (a) and (b) exhibit a prominent beating behavior, where the number and length of batter frequency are changed with *θ*_*r*_. For *θ*_*r*_ = *π*/2 (i.e., 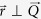), the beat frequency of 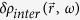 dies away and recovers the typical decaying oscillation, as shown in Fig. 5(c). Importantly, the decaying rate of all oscillations in 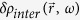, abiding by 1/*r*^2^ law regardless of *θ*_*r*_ as illustrated in the insets, always dominates over 1/*r*^3^-law decaying 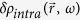 for sufficiently large *r* ≫ 1. Therefore, the battering feature in the TRS-broken WSMs is accessible in measurement of the scattering between nodes, moreover unaffected by the intranode scattering, which is important for identifying the TRS-broken WSM in the real space. Notice that this beating structure does not occur in the typical surface state of topological insulators. As far as we know, the WSM phase by breaking of time-reversal symmetry has not been yet experimentally reported and the beating feature maybe provide an alternative route to identify this new type of materials, e.g., Y_*b*_MnBi_2_.

Finally, we want to remark the influence of asymmetric coupling 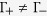 of the impurity to two Weyl nodes. From Eqs (16) and (17), one can find that the asymmetric coupling only changes quantitatively the weight between intranode scattering and internode scattering. Thus, the above obtained results are qualitatively suitable as long as we properly reset other parameters.

## Conclusions

On conclusions, we have investigated the influence of quantum impurity on the DSM and WSM materials by looking at the modification of LDOS around the impurity. It is found that the quantum impurity scattering can create the LDOS low-energy resonance, the Kondo signature, and the Friedel oscillation, all of which are sensitive to the displacement of Weyl nodes in energy or momentum. We in detail analyze the different contribution from the intra- and inter-node scattering processes and present different scenarios as a consequence of competition between them. We further study the spatial dependence of LDOS and find that the separation of Weyl nodes along with the Friedel interference oscillation leads to the unique battering feature, which arises in the intranode scattering for the IS-broken WSMs but in internode scattering for the TRS-broken WSMs. Especially, the beating feature for the TRS-broken WSMs is remarkably dependent on the spatial direction of the probing position, which is long-range measurable in real space by employing current scanning tunneling microscope technologies.

## Additional Information

**How to cite this article**: Zheng, S.-H. *et al*. Resonance states and beating pattern induced by quantum impurity scattering in Weyl/Dirac semimetals. *Sci. Rep.* **6**, 36106; doi: 10.1038/srep36106 (2016).

**Publisher’s note**: Springer Nature remains neutral with regard to jurisdictional claims in published maps and institutional affiliations.
